# Healthcare utilization among children and young people with life-limiting conditions: Exploring palliative care needs using National Health Insurance claims data

**DOI:** 10.1038/s41598-020-59499-x

**Published:** 2020-02-14

**Authors:** Cho Hee Kim, In Gyu Song, Min Sun Kim, Jin Yong Lee, Nam Gu Lim, Hee Young Shin

**Affiliations:** 1 College of Nursing, Seoul National University, Seoul, Republic of Korea; 2 National Hospice Centre, National Cancer Centre, Goyang, Republic of Korea; 3 Department of Paediatrics, Seoul National University Hospital, Seoul, Republic of Korea; 4 Department of Public Health and Community Medicine, SMG-SNU Boramae Medical Centre, Seoul, Republic of Korea; 5 Daejeon Health Institute of Technology, Daejeon, Republic of Korea; 6 Department of Health Policy and Management, Seoul National University College of Medicine, Seoul, Republic of Korea

**Keywords:** Epidemiology, Paediatric research, Palliative care

## Abstract

Paediatric palliative care (PPC) is regarded as standard care for children and young people (CYP) with life-limiting conditions (LLCs). There is a lack of knowledge about the rate of CYP with LLCs, hampering the development of PPC. This retrospective study aimed to examine population-based statistics of South Korean CYP with LLCs and the pattern of healthcare use and costs in their last year of life, analysing the National Health Insurance Service claims database for the period 2013–2015. In 2015, the number of CYP (≤24 years old) living with LLCs was 133,177, with those who died accounting for 1,032. Prevalence of LLC and mortality rate per 100,000 were highest among under-1-age group (2,151.7 and 82.7, respectively). In the last year of life, 91.8% of deceased CYP with LLCs were hospitalized at least once and the average length of stay was 101.2 days (standard deviation = 104.1). Deceased CYP with cancer spent more on healthcare than non-cancer CYP (64,266 vs. 40,694 US dollar, *p* < 0.001). The average relevance index for CYP death related to LLCs was 55.9%. Our results provide baseline information on healthcare utilization and expenditure among CYP with LLCs, which is crucial data for designing evidence-based PPC policy and services.

## Introduction

Paediatric palliative care (PPC) can be summarized as the active total care of the child’s body, mind, and spirit, which also involves providing support to the family; it is suggested to start PPC at the time of diagnosis and continue to provide throughout the course of diseases^1^. The practice of PPC started in England with the establishment of the first children’s hospice, Helen House, in 1982. In recent decades, PPC has spread worldwide, taking on various forms in different countries. For instance, the United Kingdom has many residential hospice teams and facilities, while the United States has focused on hospital-based care. Recently, the World Health Assembly announced that the provision of PPC is an ethical responsibility of the health system, and that integrating palliative care into the public healthcare system is necessary to achieve the Sustainable Development Goal 3 regarding universal health coverage^2,3^.

South Korea achieved universal health coverage in 1989^4^. The government has made efforts to improve healthcare for children, including the funding of 49 neonatal intensive care units and 7 specialized public medical centres for children^5^. Advances in medical technology have contributed to improvements in a number of health indicators, such as the infant mortality rate (2.8/1,000 live births) and childhood cancer survival rate (83.6%)^6,7^. However, PPC in Korea remains in its infancy. Over 1,000 children died from chronic complex conditions (CCCs) each year from 2005 to 2014^8^. However, there were no hospitals or institutes meeting the standards for specialized PPC in South Korea until 2015^9^. A multidisciplinary palliative care team for CYP was established at a tertiary hospital in South Korea in 2015; since then, the interest in and need for this type of care have been on the rise, and the Ministry of Health and Welfare announced its support for the development of PPC^10^.

In order to design a national PPC program, research is needed to investigate the PPC needs of patients and their families. The current study therefore aimed to examine population-based statistics of prevalence and deaths of CYP with LLCs and provide a measurement that estimates the minimum volume of CYP who may benefit from palliative care; additionally, we aimed to evaluate healthcare utilization and related expenditure among CYP with LLCs^11^.

## Methods

### Study design

A retrospective, population-level analysis was conducted using the Korean National Health Insurance Service (NHIS) claims database. This study was designed and performed in accordance with the principles of the Declaration of Helsinki. The institutional review board (IRB) of Seoul National University Hospital reviewed and exempted this study (No. 1705-002-850), as the data obtained from the NHIS were anonymized so that subjects could not be identified, and the requirement to obtain informed consent was waived.

### Data source

The NHIS has provided compulsory universal health insurance for the entire population since 2000^12^. In 2006, the NHIS database incorporated information on individuals receiving medical aid, which accounted for 2.8% of the population^13^. All NHI and medical aid claims data for insured medical services provided by all types of healthcare institutions are stored and managed in a single, centralized database, with the exception of spending on uninsured healthcare services that would be directly paid for by patients or their families^12^. The database contains information on healthcare utilization and expenditure, as well as demographic information including diagnoses assigned according to the International Statistical Classification of Disease and Related Health Problems 10^th^ revision (ICD-10). In case of death, emigration, or becoming a recipient of medical aid, eligibility for the NHI might lapse.

In order to gain access to the database, the IRB approval statement and summary of the research project was submitted to the NHIS. We accessed and analysed raw data only when based in designated research institutions. The result charts generated in the analyses were the only information allowed to be taken off the premises.

### Study population

We analysed the NHIS claims database to identify CYP aged 24 years or younger and who had used inpatient, outpatient, and/or emergency services in relation to their LLC diagnosis between January 1, 2013 and December 31, 2015. A total of 523,073 CYP with LLCs were identified. Among them, 3,995 died from their LLCs during the study period.

In order to evaluate healthcare use and costs by year and compare the annual LLCs prevalence, number of deaths, and mortality rate with other studies, we focused our analysis on CYP with LLCs who were alive or died in 2015, who were found to be 133,177 and 1,032, respectively. LLCs prevalence and mortality rates per 100,000 population by age groups and diagnostic groups were analysed using the mid-year population of 2015 as a denominator, obtained from the Korean Statistical Information Service^14^. We calculated 95% confidence intervals using a normality assumption. We divided the subjects into two disease groups, namely CYP with cancer and CYP with non-cancer LLCs, to account for distinct courses of treatment.

In order to compare the LLCs prevalence and number of deaths internationally, LLCs were defined using disease codes based on the framework of the Directory of LLCs^15,16^. Each code was categorized in accordance with diagnostic groups of CCCs^17^. Where disease codes for LLCs and CCCs matched, LLC codes were classified as the corresponding CCC category, with the remaining codes classified based on disease characteristics according to CCC categories. The list of diagnostic codes and categories was reviewed by three paediatricians and one paediatric researcher, and approved by two medical professional societies to determine the appropriateness of the final version of the codes and categories and minimize misclassification bias (see Supplementary Table S1).

### Operational definitions

Patients living with LLCs were defined as patients who were assigned LLCs disease codes for hospital admissions or outpatient department (OPD) or emergency department (ED) visits. Patients were selected if their primary and first to fifth additional diagnosis fields included one or more LLC diagnoses. Patients died with LLCs were identified as those patients who were disqualified from the NHIS because of death.

Inpatient service utilization was defined as discharge following a period of stay after admission, including same-day discharge. Length of stay was defined as the sum of hospital days. If a patient was admitted and discharged more than once during the study period, the total sum of hospital days of the multiple admissions was used. Moreover, in the case of an admission starting the previous year and finishing during the study period, the full number hospital days was used. Outpatient service utilization was defined as the use of ambulatory services without being admitted, and emergency service utilization was defined as the use of emergency services. Emergency service utilization could be underestimated, as the database does not distinguish between the use of emergency services only and the use of emergency services leading to admission; admission through ED visits was therefore categorized as inpatient service use in this study.

### Variables

The following demographic characteristics were used as independent variables: gender, four age groups (<1 year, 1–9 years, 10–19 years, and 20–24 years), area of residence (by administrative districts and metropolitan status), level of income (high, medium, and low), and type of insurance (NHI or medical aid). We could not extract information on neonates (younger than 1 month of age) because NHIS claims data only includes the age of beneficiaries in years and not in months. We included severity of disability (severe or mild to moderate) if present. According to the National Disability Registration System, the type and degree of disability are determined by specialists (paediatricians, neurologists, etc.) if the physical or mental impairment is confirmed to be fixed after treatment for 6 months; thus, CYP with rapidly progressing acute disease or children under the age of 1 are seldom classed as having a disability even if they actually do. Date of death and diagnosis determined by the ICD-10 were also extracted. We used the NHIS premium (i.e. the amount paid by the insured person to the NHIS, based on income level) as a proxy indicator of income level, which was divided into three categories: high (upper 25% premium), intermediate (middle 50% premium), and low (lower 25% premium).

### Healthcare use, expenditure, and geographic analysis

We examined average healthcare use and costs related LLCs in the last year of life for each patient in terms of the number of hospitalizations, inpatient days, OPD visits, and ED visits. In order to identify the geographic distribution of deaths and any imbalances in healthcare use regarding LLCs between 17 administrative districts, relevance indices (RIs) were calculated by dividing the number of deceased patients with LLCs who used hospitals located in the same geographic region as their residence by the total number of deceased residents with LLCs in that area^18,19^. RI was analysed based on the sum of data from 2013 to 2015, considering the small number of deceased patients with LLCs. RI is an effective indicator of the extent of patients’ healthcare use in their area of residence. A high RI represents a high number of deceased CYP with LLCs who were treated at their local hospitals in their final year of life without needing to travel to other regions to seek medical treatment.

### Statistical analysis

The data were analysed using descriptive statistics, namely means and standard deviations (SD) in the case of continuous variables, and frequencies and percentages in the case of categorical variables. Pearson’s chi-square test and analysis of variance (ANOVA) were used to evaluate the differences between cancer and non-cancer patients. All analyses were performed using SAS 9.4 (SAS Institute Inc., Cary, NC, USA) and IBM SPSS Statistics (version 20; IBM Company, Chicago, IL, USA), and the level of significance was defined as *p* < 0.05.

## Results

There were 133,177 CYP diagnosed with LLCs and who had at least one claim in 2015 (Table 1). Approximately two-thirds were older than 10 years of age, while the prevalence of LLCs was highest in the under-1 age group, with 2,151.7 per 100,000 children aged under 1 (Table 2). The majority of subjects (94.1%) were NHI beneficiaries, and 5.9% had confirmed disabilities. The average healthcare cost per patient per year was 3,372 ± 14,933 US dollar (USD) (Table 3). Among all subjects, cancer patients were admitted for longer periods than non-cancer patients (30.7 vs. 25.7 days per year, *p* < 0.001) and visited the ED more often (1.6 vs. 1.2 visits per year, *p* < 0.001). The number of OPD visits was higher among non-cancer patients than cancer patients (9.5 vs 11.4 visits per year, *p* < 0.001). Detailed healthcare use and costs are presented in Supplementary Table S2.

**Table 1:** Demographic characteristics of children and young people with life-limiting conditions, 2015.

Variables	Total, n (%)	Cancer, n (%)	Non-cancer, n (%)	*p*-value
Total	133,177	34,943 (26.2)	98,234 (73.8)	
Gender				<0.001
Male	71,764 (53.9)	17,121 (49.0)	54,643 (55.6)	
Female	61,413 (46.1)	17,822 (51.0)	43,591 (44.4)	
Age group (year)				<0.001
<1	9,084 (6.8)	363 (1.0)	8,721 (8.9)	
1–9	35,247 (26.5)	5,743 (16.4)	29,504 (30.0)	
10–19	49,120 (36.9)	13,987 (40.0)	35,133 (35.8)	
20–24	39,726 (29.8)	14,850 (42.5)	24,876 (25.3)	
Type of insurance qualification				<0.001
National Health Insurance	125,289 (94.1)	33,304 (95.3)	91,985 (93.6)	
Medical aid	7,888 (5.9)	1,639 (4.7)	6,249 (6.4)	
Level of income^a^				<0.001
High	46,137 (34.6)	12,750 (36.5)	33,387 (34.0)	
Medium	53,547 (40.2)	13,405 (38.4)	40,142 (40.9)	
Low	33,493 (25.1)	8,788 (25.1)	24,705 (25.1)	
Severity of disability^b^				<0.001
Severe	17,532 (13.2)	1,452 (4.2)	16,080 (16.4)	
Mild to moderate	3,618 (2.7)	1,140 (3.3)	2,478 (2.5)	
Without confirmed disability	112,027 (84.1)	32,351 (92.6)	79,676 (81.1)	
Diagnostic category^c^				
Malignancy		34,943 (26.2)		
Neurologic & neuromuscular			29,106 (21.9)	
Cardiovascular			20,483 (15.4)	
Renal & urologic			19,595 (14.7)	
Hematologic or immunologic			12,716 (9.5)	
Gastrointestinal			11,732 (8.8)	
Respiratory			8,976 (6.7)	
Other congenital or genetic defect			7,778 (5.8)	
Metabolic			7,505 (5.6)	
Premature & neonatal			5,946 (4.5)	
Miscellaneous			511 (0.4)	
Area of residence by administrative district				<0.001
Seoul, Gyeonggi, Incheon	80,996 (60.8)	22,415 (64.1)	58,581 (59.6)	
Gangwon	2,580 (1.9)	615 (1.8)	1,965 (2.0)	
Chungcheong	11,458 (8.6)	2,970 (8.5)	8,488 (8.6)	
Daegue, Gyeongbuk	9,377 (7.0)	1,815 (5.2)	7,562 (7.7)	
Busan, Ulsan Gyeongnam	17,288 (13.0)	4,331 (12.4)	12,957 (13.2)	
Jeolla	10,018 (7.5)	2,494 (7.1)	7,524 (7.7)	
Jeju	1,460 (1.1)	303 (0.9)	1,157 (1.2)	
Area of residence				0.058
Metropolitan	60,322 (45.3)	15,676 (44.9)	44,646 (45.4)	
Non-metropolitan	72,855 (54.7)	19,267 (55.1)	53,588 (54.6)	

^a^Level of income was categorized based on patients’ NHIS premium as high level (upper 25% premium), intermediate level (middle 50%), and low level (lower 25%).

^b^Severity of disability was divided according to their disability grade into severe (grade 1–3) and mild (grade 4–6).

^c^Values are not mutually exclusive.

**Table 2:** Prevalence of life-limiting conditions and mortality rate (per 100,000 population) among children and young people aged 0 to 24 years by age group, 2015.

Age group (years)	Prevalence of life-limiting conditions	95% CI	Mortality rate	95% CI
Total	955.1	(950.0, 960.2)	9.3	(8.8, 9.9)
<1	2,151.7	(2,107.7, 2,196.4)	82.7	(74.2, 91.8)
1–9	844.8	(836.0, 853.7)	6.1	(5.3, 6.9)
10–19	841.1	(833.7, 848.6)	6.2	(5.5, 6.8)
20–24	1,132.0	(1,120.9, 1,143.2)	9.7	(8.7, 10.)

CI: confidence interval.

**Table 3:** Patterns of healthcare utilization and expenditure of children and young people with life-limiting conditions by diagnostic group, 2015.

Variables	Total, mean ± SD	Cancer, mean ± SD	Non-cancer, mean ± SD	*p*-value
Total, n (%)	133,177 (100.0)	34,943 (26.2)	98,234 (73.8)	
Total expenditure	3,372 ± 14,933	4,321 ± 14,234	3,034 ± 15,160	<0.001
**Inpatient service**				
Length of stay (days)	27.2 ± 51.5	30.7 ± 51.0	25.7 ± 51.7	<0.001
Inpatient expenditure	2,373 ± 10,647	3,362 ± 13,304	2,021 ± 9,499	<0.001
**Outpatient service**				
Per capita OPD visits	10.9 ± 25.9	9.5 ± 19.0	11.4 ± 28.1	<0.001
Outpatient expenditure	985 ± 10,077	946 ± 2,522	999 ± 11,637	0.183
**Emergency service**				
Per capita ED visits	1.2 ± 1.9	1.6 ± 1.9	1.2 ± 1.9	<0.001
Emergency expenditure	14 ± 182	13 ± 80	15 ± 206	0.004

SD: standard deviation; OPD: outpatient department; ED: emergency department

All expenditure values are in 2019 US dollar: 1 USD = 1,121.10 KRW.

The total number of CYP deaths in the 2015 cohort was 1,302, which is 9.3 deaths in 100,000 CYP (Table 4). Of these, 761 patients (58.4%) were male, and 349 (26.8%) were younger than 1 year. Among age groups, the number of deaths was highest at 360 (27.6%) in the 10-19-year-old group, while the highest mortality rate (82.7 deaths per 100,000) was found in the under-1 group. Compared to cancer deaths, non-cancer deaths were more frequent in patients aged under 1 (39.1% vs. 4.1%, *p* < 0.001). NHI beneficiaries accounted for 90.3% of the total CYP deaths. The proportion of deceased patients who had severe disabilities was higher in the non-cancer group than the cancer group (31.6% vs. 11.1%, *p* < 0.001).

**Table 4:** Demographic characteristics of deceased children and young people with life-limiting conditions, 2015.

Variables	Total, n (%)	Cancer, n (%)	Non-cancer, n (%)	*p*-value
Total	1,302 (100.0)	458 (35.2)	844 (64.8)	
Gender				0.208
Male	761 (58.4)	257 (56.1)	504 (59.7)	
Female	541 (41.6)	201 (43.9)	340 (40.3)	
Age of death (year)				<0.001
<1	349 (26.8)	19 (4.1)	330 (39.1)	
1–9	253 (19.4)	102 (22.3)	151 (17.9)	
10–19	360 (27.6)	168 (36.7)	192 (22.7)	
20–24	340 (26.1)	169 (36.9)	171 (20.3)	
Type of insurance qualification				0.001
National Health Insurance	1,176 (90.3)	431 (94.1)	745 (88.3)	
Medical aid	126 (9.7)	27 (5.9)	99 (11.7)	
Level of income^a^				0.075
High	365 (28.0)	137 (29.9)	228 (27.0)	
Medium	564 (43.3)	179 (39.1)	385 (45.6)	
Low	373 (28.6)	142 (31.0)	231 (27.4)	
Severity of disability^b^				<0.001
Severe	318 (24.4)	51 (11.1)	267 (31.6)	
Mild to moderate	30 (2.3)	20 (4.4)	10 (1.2)	
Without confirmed disability	954 (73.3)	387 (84.5)	567 (67.2)	
Diagnostic category^c^				
Malignancy		458 (35.2)		
Neurologic & neuromuscular			397 (30.5)	
Cardiovascular			262 (20.1)	
Renal & urologic			324 (24.9)	
Hematologic or immunologic			307 (23.6)	
Gastrointestinal			72 (5.5)	
Respiratory			204 (15.7)	
Other congenital or genetic defect			88 (6.8)	
Metabolic			132 (10.1)	
Premature & neonatal			97 (7.5)	
Miscellaneous			57 (4.4)	
Area of residence by administrative districts				0.043
Seoul, Gyeonggi, Incheon	741 (56.9)	289 (63.1)	452 (53.6)	
Gangwon	26 (2.0)	8 (1.7)	18 (2.1)	
Chungcheong	111 (8.5)	31 (6.8)	80 (9.5)	
Daegue, Gyeongbuk	109 (8.4)	28 (6.1)	81 (9.6)	
Busan, Ulsan, Gyeongnam	184 (14.1)	60 (13.1)	124 (14.7)	
Jeolla	114 (8.8)	37 (8.1)	77 (9.1)	
Jeju	17 (1.3)	5 (1.1)	12 (1.4)	
Area of residence				0.313
Metropolitan	596 (45.8)	201 (43.9)	395 (46.8)	
Non-metropolitan	706 (54.2)	257 (56.1)	449 (53.2)	

^a^Level of income was categorized based on patients’ NHIS premium as high level (upper 25% premium), intermediate level (middle 50%), and low level (lower 25%).

^b^Severity of disability was divided according to their disability grade into severe (grade 1–3) and mild (grade 4–6).

^c^Values are not mutually exclusive.

### Healthcare utilization and expenditure

We examined healthcare use and costs during the last year of patients’ lives (Table 5). Among CYP with LLCs who died in 2015, the proportion of patients who used inpatient, outpatient, or emergency services at least once in their last year of life were 91.8%, 83.5%, and 39.9%, respectively. Total mean healthcare expenditure was 48,986 ± 59,084 USD per patient. Deceased CYP with cancer spent more on total healthcare than deceased patients with non-cancer LLCs (64,266 vs. 40,694 USD, *p* < 0.001). The deceased patients with cancer stayed in hospital longer (130.7 vs. 83.5 days, *p* < 0.001) and spent more on inpatient services (60,397 vs. 39,141 USD, *p* < 0.001). The average number of OPD visits was higher in the cancer group (46.4 vs. 36.1, *p* < 0.001). The average number of ED visits was 2.4 for cancer patients and 2.2 for non-cancer patients, but the difference was not statistically significant (*p* = 0.675) (Table 5).

**Table 5:** Patterns of healthcare utilization and expenditure in the year prior to death among deceased children and young people aged 0 to 24 years with life-limiting conditions, 2015.

Variables	Total, mean ± SD	Cancer, mean ± SD	Non-cancer, mean ± SD	*p*-value
Total	1,302 (100.0)	458 (35.2)	844 (64.8)	
Total expenditure	48,986 ± 59,084	64,266 ± 58,165	40,694 ± 57,947	<0.001
**Inpatient service**				
≥1 Hospitalization per year, n (%)^a^	1,195 (91.8)	449 (98.0)	746 (88.4)	
Length of stay (days)	101.2 ± 104.0	130.7 ± 97.3	83.5 ± 104.0	<0.001
Inpatient expenditure	46,618 ± 57,935	60,397 ± 57,845	39,141 ± 56,630	<0.001
**Outpatient service**				
≥1 OPD visit per year, n (%)^b^	1,087 (83.5)	451 (98.5)	636 (75.4)	
Per capita OPD visits	40.4 ± 40.3	46.4 ± 32.4	36.1 ± 44.5	<0.001
Outpatient expenditure	2,220 ± 7,035	3,738 ± 4,692	1,396 ± 7,907	<0.001
**Emergency service**				
≥1 ED visit per year, n (%)^c^	521 (39.9)	201 (43.9)	320 (37.9)	
Per capita ED visits	2.3 ± 5.0	2.4 ± 4.1	2.2 ± 5.5	0.675
Emergency expenditure	148 ± 346	131 ± 363	157 ± 336	0.212

SD: standard deviation; OPD: outpatient department; ED: emergency department. All expenditure values are in 2019 US dollar: 1 USD = 1,121.10 KRW.

^a^No. of patients who were ever hospitalized.

^b^No. of patients who ever visited OPD.

^c^No. of patients who ever visited ED.

Total mean healthcare cost was highest in patients younger than 1 year (49,839 ± 52,879 USD), followed by those between 1 and 9 years old (44,998 ± 48,769 USD); this figure continued to decrease as age increased. The average length of stay and number of OPD visits per patient was highest in the 1-9-year-old group (112.5 ± 105.8 and 58.8 ± 46.6 days, respectively) (see Supplementary Table S3). Based on the level of income, significant differences in both expenditure on inpatient services and total healthcare services showed a similar tendency, with the high-income group spending the most, and healthcare spending decreased with increasing income. Moreover, spending on outpatient services was the highest in the low-income group (2,682 ± 9,816 USD, *p* < 0.001) (see Supplementary Table S4).

### Relevance index

**Figure 1:**
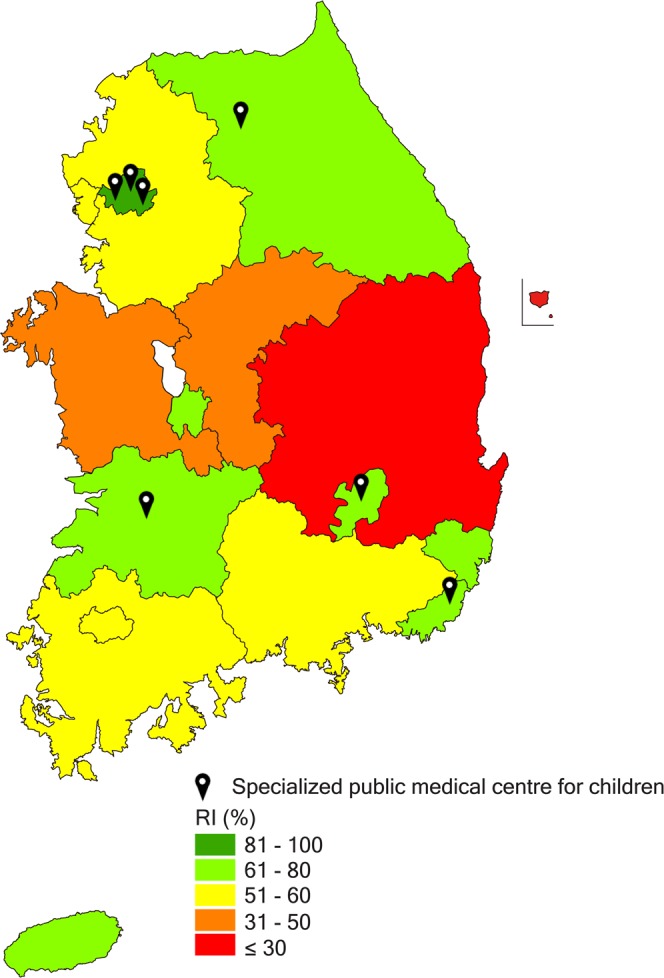
Relevance indices (RI) for deceased children and young people with life-limiting conditions between 2013 and 2015 and specialized public medical centres for children in Korea. This map was modified from https://commons.wikimedia.org/wiki/File:Largest_religion_by_province_in_South_Korea.svg using Adobe Illustrator CC ver. 24.0.1 (https://www.adobe.com/products/illustrator.html).

The total cumulative number of deceased CYP with LLCs between 2013 and 2015 was 3,995. The average RI was 55.9% and ranged from 28.8% to 89.6%, excluding Sejong, where no LLC-related deaths of CYPs were reported (Fig. 1). The details of the regional distribution of deaths and healthcare use of CYP with LLCs can be found as Supplementary Table S5. Overall, RI was higher in metropolitan areas with specialized public medical centres for children, such as Seoul (89.6%), Daegu (74.2%), and Busan (69.6%), demonstrating high rates of utilization in these areas. Jeju, which is an island, also showed a high RI of 72.5%. On the contrary, the RI was low for Gyeongbuk (28.8%), Chungnam (35.3%), and Chungbuk (36.2%).

## Discussion

Using National Health Insurance claims data, we investigated the needs of CYP with LLCs including those who died in order to establish a national PPC system.

The prevalence of LLCs per 100,000 population (955.1) was higher than that in a previous study in England, but similar to the prevalence reported in Scotland, which included outpatients in the study group as done in this study (Table 6)^15,20^. Cancer was the most prevalent LLC (26.2%), with the remaining three-quarters of patients having non-cancer LLCs. In the non-cancer group, neurologic and neuromuscular LLCs were the most common diagnostic group, and cardiovascular and renal diseases followed (Tables 1 and 2). Policy for hospice and palliative care in South Korea has focused only on adult cancer patients^21^. However, our results regarding LLCs prevalence and mortality rates indicate that PPC policy should encompass not only patients with cancer but also those with non-cancer LLCs. In contrast to previous studies, the cardiovascular group ranked high, and the congenital/genetic defect group ranked low^15,20,22^. This may be because we categorized disease codes in accordance with diagnostic groups of CCCs; thus, some congenital diseases were classified in organ-specific disease groups^17^. Although the prevalence of the congenital disease group was low, its highest prevalence was in the under-1 age group, and this trend was similar to that found in a previous study (Table 6)^20^.

**Table 6:** Prevalence of life-limiting conditions and mortality rate (per 100,000 population) of children and young people aged 0 to 24 years by diagnostic group, 2015.

Diagnostic group	Prevalence of life-limiting conditions	95% CI	Mortality rate	95% CI
Total	955.1	(950.0, 960.2)	9.3	(8.8, 9.9)
Malignancy	250.6	(248.0, 253.2)	3.3	(3.0, 3.6)
Neurologic & neuromuscular	208.7	(206.3, 211.1)	2.8	(2.6, 3.1)
Cardiovascular	146.9	(144.9, 148.9)	1.9	(1.7, 2.1)
Renal & urologic	140.5	(138.6, 142.5)	2.3	(2.1, 2.6)
Hematologic or immunologic	91.2	(89.6, 92.8)	2.2	(2.0, 2.5)
Gastrointestinal	84.1	(82.6, 85.7)	0.5	(0.4, 0.7)
Respiratory	64.4	(63.0, 65.7)	1.5	(1.3, 1.7)
Other congenital or genetic defect	55.8	(54.5, 57.0)	0.6	(0.5, 0.8)
Metabolic	53.8	(52.6, 55.1)	0.9	(0.8, 1.1)
Premature & neonatal	42.6	(41.6, 43.7)	0.7	(0.6, 0.8)
Miscellaneous	3.7	(3.4, 4.0)	0.4	(0.3, 0.5)

CI: confidence interval.

The average number of days spent in hospital in our sample was higher than that found in previous studies^23,24^. Access to health services is high in South Korea, as the country achieved universal health coverage and has a higher number of hospital beds than other developed countries. This may lead to longer hospital stays and more hospital visits relative to other countries^25^. This fact combined with the earlier finding that the most common location of paediatric CCC-related deaths was the hospital (85.5%)^8^ reveals the need for palliative care in hospital settings. Implementing both a general palliative care approach and specialized palliative care in inpatient settings is required to integrate palliative care into disease-modifying treatments for CYP with LLCs.

The mean annual total cost of healthcare for CYP with LLCs who lived or died in 2015 (3,372 ± 14,933 and 48,986 ± 59,084 USD, respectively) was considerably higher than that of general CYP (1,109 USD)^26^. The mean annual total cost of inpatient services in the last year of life accounted for 95.2% of the total cost—lower than that found in earlier international studies^27,28^. We also found a difference between the two diagnostic groups, as expenditure was higher for cancer patients than non-cancer patients (64,266 ± 58,165 vs. 40,694 ± 57,947 USD, respectively, *p* < 0.001). In 2015, the co-payment rate of cancer patients was 5%, in contrast to the 10–20% for inpatient services and maximum 70% for outpatient services among non-cancer patients. Although there were upper limits on yearly medical expenses (1,000 to 4,200 USD according to family income), cancer patients may have paid less than non-cancer patients because of the lower co-payment rate. These differences in health policy and treatment strategies (scheduled chemotherapy in cancer patients) might affect healthcare utilization patterns and expenditure.

According to the Act on Welfare of Persons with Disabilities, individuals with disabilities and their families qualify for financial aid, activity-supporting assistance services, and other benefits if their disability has been confirmed^29^. While 15.9% of the subjects in this study had disabilities, the majority did not qualify for these benefits (Table 1). Moreover, only children with disabilities over the age of 6 qualify for activity-supporting assistance services^29^. While we can infer that this leads to unmet needs in care services among CYP with LLCs and their families, the nature of these require further investigation.

Most regions where the RI of CYP deaths was less than 60% did not have specialized public medical centres for children. Patients and families in these regions may spend children’s end-of-life period far away from their home. Thus, these individuals require the development of community-based hospice and palliative care for children, or liaison with home hospice care or hospice inpatient facilities for adults. Moreover, the additional designation of specialized public medical centres for children in these regions is needed.

The main strength of our study is that, to the best of our knowledge, it is the first to evaluate healthcare utilization and expenditure among CYP with LLCs in South Korea. Currently, there are limited data that can be used to assess healthcare utilization of severely ill paediatric patients. Our study addresses this gap. Furthermore, we used population-level data, encompassing both outpatient and inpatient services, which contributes to the robustness of our findings. Lastly, this study describes a method for developing and implementing PPC from a public healthcare perspective, using NHIS claims data.

An important limitation to our study is that it did not consider out-of-pocket costs, as the NHIS cannot collect these data. However, the NHIS data have been widely used for evaluating needs or outcomes of health policies in South Korea^30,31^. Second, we could not access information on location of death within hospitals (e.g. intensive care unit or general wards) or life-sustaining treatments, which could have added more depth to our results. In order to analyse the effect of PPC, further studies are needed. Third, in order to compare data internationally and secure reproducibility, we used disease codes based on the framework of the Directory of LLCs^15,16^. Therefore, patients who did not receive these codes were not included in this study; thus, the number of patients who need palliative care may be different in reality.

The results of the current study provided preliminary data contributing to the design of a government-funded PPC pilot program, into which patients with LLCs (both cancer and non-cancer) aged 24 and younger have been enrolled. The pilot program has been designed to support the establishment of specialized paediatric palliative consulting teams in tertiary hospitals. This program was launched in two hospitals in July 2018 and two more in January 2019. Each hospital is funded with 160,000 USD per year, and patients do not need to make additional payments for palliative care. As healthcare professionals in South Korea are unfamiliar with PPC, we used the Paediatric Palliative Screening Scale from Switzerland to assist them in deciding when is the best time to introduce PPC to patient and family^32^. Furthermore, unmet needs should be assessed such as the need for community-based PPC, to expand the PPC program appropriately. Also, to improve accessibility, the PPC program is needed to be implemented in other specialized public medical centres for children, as well as tertiary hospitals that are not designated as specialized public medical centres for children, considering the distribution of residences of CYP with LLCs.

Palliative care has become an essential aspect of paediatrics, and the number of countries that provide PPC has increased^2,9^. CYP who require palliative care need more consideration, and policy development should be based on their needs. Countries planning to set up a PPC system should first identify the characteristics, distribution, and needs of CYP with LLCs. The analysis of national health data can be an efficient way to assess such aspects and may help to establish socially and culturally appropriate PPC systems worldwide.

## Supplementary information

Supplementary information.

## Data Availability

The NHIS database is operated by the Korean National Health Insurance Sharing Service. To protect privacy and public interests, access to the data is available only in designated research centres in South Korea. Additional data to support the review has been submitted as supplementary data. Further analyses are being conducted using this dataset.
