# Author Correction: Healthcare utilization among children and young people with life-limiting conditions: Exploring palliative care needs using National Health Insurance claims data

**DOI:** 10.1038/s41598-020-69836-9

**Published:** 2020-08-11

**Authors:** Cho Hee Kim, In Gyu Song, Min Sun Kim, Jin Yong Lee, Nam Gu Lim, Hee Young Shin

**Affiliations:** 1 College of Nursing, Seoul National University, Seoul, Republic of Korea; 2 National Hospice Centre, National Cancer Centre, Goyang, Republic of Korea; 3 Department of Paediatrics, Seoul National University Hospital, Seoul, Republic of Korea; 4 Department of Public Health and Community Medicine, SMG-SNU Boramae Medical Centre, Seoul, Republic of Korea; 5 Daejeon Health Institute of Technology, Daejeon, Republic of Korea; 6 Department of Health Policy and Management, Seoul National University College of Medicine, Seoul, Republic of Korea

Correction to: *Scientific Reports* 10.1038/s41598-020-59499-x, published online 14 February 2020

The Results section under the subheading ‘Relevance index’ contains an error where

“On the contrary, the RI was low for Gyeongbuk (28.8%), Chungnam (35.3%), and Chungbuk (36.2%).”

should read:

“On the contrary, the RI was low for Gyeongbuk (28.8%), Chungnam (35.3%), and Gyeonggi (43.9%).”

Figure 1 also contains an error where the location of Gyeonggi was incorrectly coloured yellow instead of orange. The correct Figure 1 appears below.

**Figure 1:**
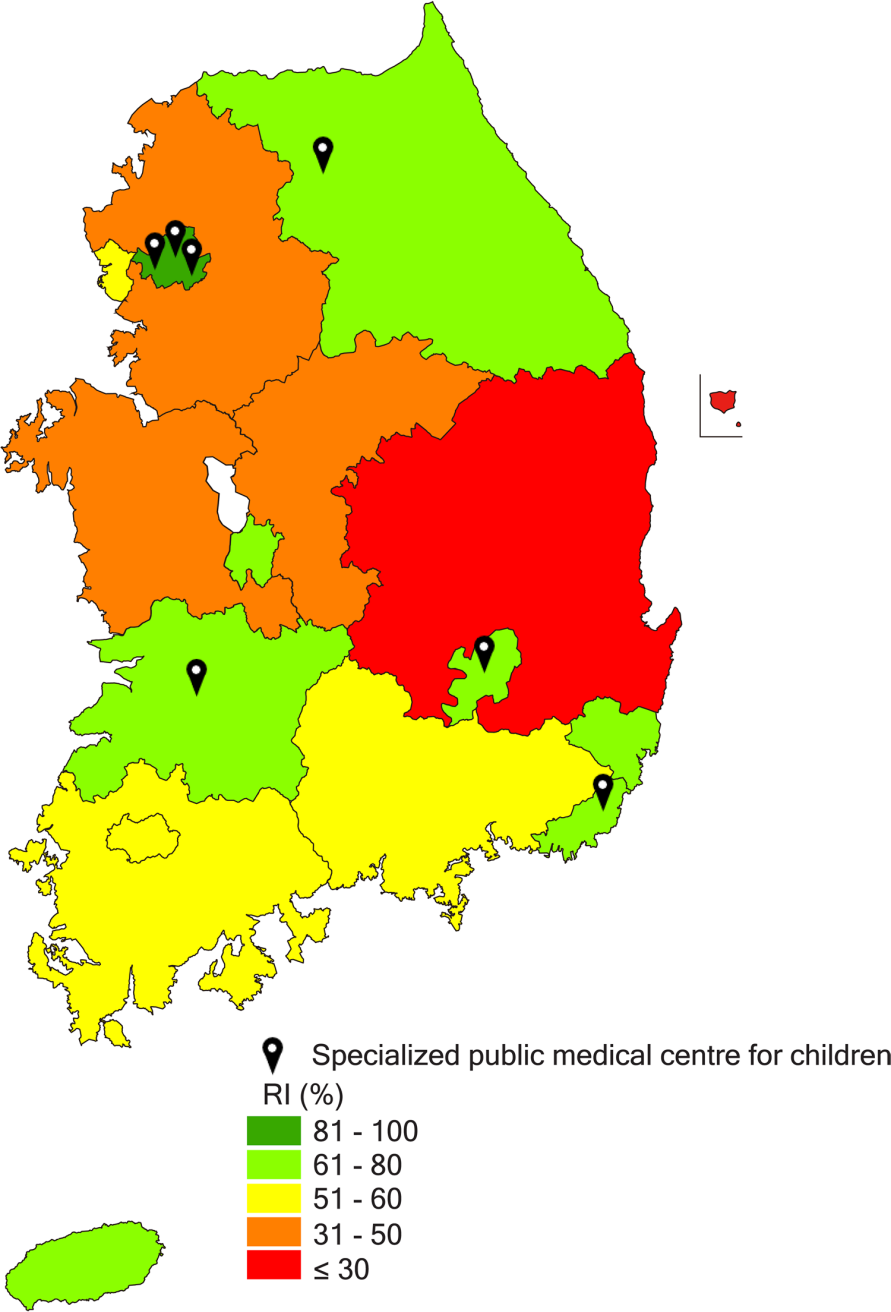
Relevance indices (RI) for deceased children and young people with life-limiting conditions between 2013 and 2015 and specialized public medical centres for children in Korea. This map was modified from https://commons.wikimedia.org/wiki/File:Largest_religion_by_province_in_South_Korea.svg using Adobe Illustrator CC ver. 24.0.1 (https://www.adobe.com/products/illustrator.html).

The Acknowledgements section in this Article is incomplete.

“This study was funded by a grant from the National R&D Program for Cancer Control, Ministry of Health and Welfare, Republic of Korea (1631173). The funders had no role in the study design, data collection and analysis, data interpretation, writing the manuscript, or publication. The abstract was presented at the 4th Global Gathering Maruzza Congress on Paediatric Palliative Care and was awarded the First Prize of No Pain for Children Award for Excellence in Paediatric Palliative Care.”

should read:

“This study was funded by a grant from the National R&D Program for Cancer Control, Ministry of Health and Welfare, Republic of Korea (1631173). This study used NHIS-NHID (National Health Information Database) data (NHIS-2017-1-174) made by NHIS. The funders nor NHIS had no role in the study design, data collection, and analysis, data interpretation, writing the manuscript, or publication. The abstract was presented at the 4th Global Gathering Maruzza Congress on Paediatric Palliative Care and was awarded the First Prize of No Pain for Children Award for Excellence in Paediatric Palliative Care.”

