# Supplementary material for: Healthcare utilization among children and young people with life-limiting conditions: Exploring palliative care needs using National Health Insurance claims data
Source: Sci Rep. 2020 Feb 14;10:2692. doi: 10.1038/s41598-020-59499-x (PMC7021730; doi:10.1038/s41598-020-59499-x)
Supplement: Supplementary file 1 — Supplementary information. [file 41598_2020_59499_MOESM1_ESM.pdf]

Healthcare utilization among children and young people with life-limiting conditions: Exploring palliative care needs using National Health Insurance claims data

Authors

Kim, Cho Hee [1†], Song, In Gyu[2†], Kim, Min Sun[3*], Lee, Jin Yong[4], Lim, Nam Gu[5], Shin, Hee Young[3]

1. College of Nursing, Seoul National University, Seoul, Republic of Korea
2. National Hospice Centre, National Cancer Centre, Goyang, Republic of Korea
3. Department of Paediatrics, Seoul National University Hospital, Seoul, Republic of Korea
4. Department of Public Health and Community Medicine, SMG-SNU Boramae Medical Centre, Seoul, Republic of Korea
5. Daejeon Health Institute of Technology, Daejeon, Republic of Korea

Corresponding author:

Kim, Min Sun, MD

Seoul National University

101, Daehak-ro, Jungno-gu, Seoul 03080, Republic of Korea

Mobile: +82-10-2724-5345

Tel: +82-2-2072-1756

E-mail: singrumi@gmail.com

[†] These authors contributed equally to this study and should be considered co-first authors

Supplementary Table S1. Categories of CCCs and the corresponding LLCs diagnoses

**Categories of CCCs**			**Corresponding LLCs Diagnosis**
**Categories**	**Subcategories**	**Diagnosis and Procedure codes**	**ICD-10 (KCD-6)**
**Neurologic & neuromuscular**	Brain and spinal cord malformations	Q00-Q07, G90.1	A17, A81.0, A81.1,<br>F80.3, F84.2,<br>G10, G11.1, G11.3, G12, G20, G23.0, G23.8, G31.8, G31.9, G35, G40.3,<br>G40.4, G40.5, G60.0, G60.1, G70.2,<br>G70.9, G71.0, G71.1, G71.2, G71.3,<br>G80, G80.8, G82.3, G82.4, G82.5,<br>G93.4, G93.6, G93.7,<br>I613,<br>Q00.0, Q01, Q03.1, Q03.9, Q04.0,<br>Q04.2, Q04.3, Q04.4, Q04.6, Q04.9,<br>Q07.0, Q85.1
	Mental retardation	F71-F73	
	CNS degeneration and diseases	E75.0, E75.1, E75.2, E75.4, F84.2, G11.1-G11.4, G11.8, G11.9, G12.0- G12.2, G12.8, G12.9, G31.00, G31.08, G31.8, G31.80, G31.81, G31.82, G31.88, G32.8, G93.8, G93.9, G94, G91.1, G31.9, G25.3, G95.1, G95.8, G90.9, Q85.1	
	Infantile cerebral palsy	G80	
	Epilepsy	G40.311, G40.301, G40.211, G40.219, G40.411, G40.419, G40.111, G40.119, G40.804, G40.911, G40.919	
	Other disorders of CNS	G37.1, G37.2, G37.8, G81.90, G82.90, G82.50-G82.54, G83.5, G83.9, G93.1, G93.5, R40.3, 0016070, 0016071, 0016072, 0016073, 0016074, 0016075, 0016076, 0016077, 0016078, 001607B, 0016370, 0016371, 0016372, 0016373, 0016374, 0016375, 0016376, 0016377, 0016378, 001637B, 001U074, 001U076, 001U077, 001U079, 001U374, 001U376, 001U377, 001U379, 00B70ZZ, 00B73ZZ, 00B74ZZ, 00T70ZZ, 00T73ZZ, 00T74ZZ	
	Occlusion of cerebral arteries	I63.30, I63.50	
	Muscular dystrophies and myopathies	G71, G72	
	Movement diseases	G10, G20, G21.0, G21.11, G21.19, G21.8, G23.0-G23.2, G23.8, G24.02, G24.8, G25.3-G25.5, G25.81-G25.83, G25.89, G25.9, G80.3	
	Devices	T85.09XA, T85.190A, T85.192A, T85.199A, T85.79XA, Z98.2, Z45.41, Z45.42, 00160J0, 00160J1, 00160J2, 00160J3, 00160J4, 00160J5, 00160J6, 00160J7, 00160J8, 00160JB, 00160K0, 00160K1, 00160K2, 00160K3, 00160K4, 00160K5, 00160K6, 00160K7, 00160K8, 00160KB, 00163J0, 00163J1, 00163J2, 00163J3, 00163J4, 00163J5, 00163J6, 00163J7, 00163J8,	N/A

Categories of CCCs			Corresponding LLCs Diagnosis
Categories	Subcategories	Diagnosis and Procedure codes	ICD-10 (KCD-6)
		00163JB, 00163K0, 00163K1, 00163K2, 00163K3, 00163K4, 00163K5, 00163K6, 00163K7, 00163K8, 00163KB, 001U0J4, 001U0J6, 001U0J7, 001U0J9, 001U0K4, 001U0K6, 001U0K7, 001U0K9, 001U3J4, 001U3J6, 001U3J7, 001U3J9, 001U3K4, 001U3K6, 001U3K7, 001U3K9, 009600Z, 009630Z, 009640Z, 00H00MZ, 00H03MZ, 00H04MZ, 00H60MZ, 00H63MZ, 00H64MZ, 00HE0MZ, 00HE3MZ, 00HE4MZ, 00HU0MZ, 00HU3MZ, 00HU4MZ, 00HV0MZ, 00HV3MZ, 00HV4MZ, 00W60JZ, 00W63JZ, 00W64JZ, 00WU0JZ, 00WU3JZ, 00WU4JZ, 01HY0MZ, 01HY3MZ, 01HY4MZ, 0DH60MZ, 0DH63MZ, 0DH64MZ, 0W110J9, 0W110JB, 0W110JG, 0W110JJ, 3E1Q38X, 3E1Q38Z	
	Transplantation	N/A	N/A
**Cardiovascular**	Heart and great vessel malformations	Q20, Q21.2-Q24, Q25.1-Q26, Q28.2, Q28.3, Q28.9, 02170ZP, 02170ZQ, 02170ZR, 02BK0ZZ, 02LR0ZT, 02LS0ZZ, 02LT0ZZ, 02NH0ZZ, 02RK0JZ, 02RL0JZ, 02RM0JZ, 02RP0JZ, 02RQ07Z, 02RQ0JZ, 02RR07Z, 02RR0JZ, 02SP0ZZ, 02SW0ZZ, 02U70JZ, 02UA0JZ, 02UA3JZ, 02UA4JZ, 02VR0ZT, 02WA0JZ	I21, I27.0, I42, I81,<br>P29.0, P29.3,<br>Q20.0, Q20.3, Q20.4, Q20.6, Q20.8,<br>Q21.3, Q21.8, Q22.0, Q22.1, Q22.4,<br>Q22.5, Q22.6, Q23.0, Q23.2, Q23.4,<br>Q23.9, Q25.4, Q25.6, Q26.2, Q26.4,<br>Q26.8, Q28.2,<br>T86.2
	Endocardium diseases	I34.0, I34.8, I36.0, I36.8, I37.0, I37.8	
	Cardiomyopathies	I42, I43, I51.5	
	Conduction disorder	I44, I45, I47, I48, I49.0	
	Dysrhythmias	I49.1-I49.5, I49.8, I49.9, R00.1	
	Other	I27.0, I27.1, I27.2, I27.81, I27.89, I27.9, I50.9, I51.7, I51.81, I63.139, I63.239, Z95.1	
	Devices	T82.519A, T82.529A, T82.539A, T82.599A, T82.110A, T82.111A, T82.120A, T82.121A, T82.190A, T82.191A, T82.01XA, T82.02XA, T82.03XA, T82.09XA, T82.211A, T82.212A, T82.213A, T82.218A, T82.221A, T82.222A, T82.223A, T82.228A, T82.518A, T82.528A, T82.538A, T82.598A, T82.6XXA, T82.7XXA, Z95.0, Z95.2, Z95.3, Z95.810-Z95.812, Z95.818,	N/A

Categories of CCCs			Corresponding LLCs Diagnosis ICD-10 (KCD-6)
Categories	Subcategories	Diagnosis and Procedure codes	
		Z45.010, Z45.018, Z45.02, Z45.09, Z95.9, 02H40JZ, 02H40KZ, 02H43JZ, 02H44JZ, 02H44KZ, 02H60JZ, 02H60KZ, 02H63JZ, 02H63KZ, 02H63MZ, 02H64JZ, 02H64KZ, 02H70KZ, 02H73JZ, 02H73KZ, 02H73MZ, 02H74KZ, 02HA0QZ, 02HA0RS, 02HA0RZ, 02HA3QZ, 02HA3RS, 02HA4QZ, 02HA4RS, 02HK0JZ, 02HK0KZ, 02HK3JZ, 02HK3KZ, 02HK3MZ, 02HK4JZ, 02HK4KZ, 02HL0JZ, 02HL0KZ, 02HL0MZ, 02HL3JZ, 02HL3KZ, 02HL3MZ, 02HL4JZ, 02HL4KZ, 02HL4MZ, 02HN0JZ, 02HN0KZ, 02HN0MZ, 02HN3JZ, 02HN3KZ, 02HN3MZ, 02HN4JZ, 02HN4KZ, 02HN4MZ, 02WA0QZ, 02WA0RZ, 02WA3QZ, 02WA3RZ, 02WA4QZ, 02WA4RZ, 03HK0MZ, 03HK3MZ, 03HK4MZ, 03HL0MZ, 03HL3MZ, 03HL4MZ, 03WY0MZ, 03WY3MZ, 03WY4MZ, 0JH600Z, 0JH605Z, 0JH606Z, 0JH607Z, 0JH608Z, 0JH609Z, 0JH60AZ, 0JH60MZ, 0JH60PZ, 0JH630Z, 0JH635Z, 0JH636Z, 0JH637Z, 0JH638Z, 0JH639Z, 0JH63AZ, 0JH63MZ, 0JH63PZ, 0JH70MZ, 0JH73MZ, 0JH800Z, 0JH805Z, 0JH806Z, 0JH807Z, 0JH808Z, 0JH809Z, 0JH80AZ, 0JH80MZ, 0JH80PZ, 0JH830Z, 0JH835Z, 0JH836Z, 0JH837Z, 0JH838Z, 0JH839Z, 0JH83AZ, 0JH83MZ, 0JH83PZ, 0JWT0MZ, 0JWT0PZ, 0JWT3MZ, 0JWT3PZ, 0JWTXMZ, 4B02XSZ, 4B02XTZ, 5A02110, 5A02116, 5A0211D, 5A02210, 5A02216, 5A0221D	
	Transplantation	T86.20-T86.22, Z94.1, 02YA0Z0, 02YA0Z1, 02YA0Z2	N/A
Respiratory	Respiratory malformations	Q30-Q34, P280	E84,
	Chronic respiratory diseases	G47.35, I27.82, I43, J84.112, J96.20, Z90.2	J84.1, J96, J98.4,
	Cystic fibrosis	E84	Q32.1, Q33.6,
	Other	0B110Z4, 0B113Z4, 0B114Z4, 0BTC0ZZ, 0BTC4ZZ, 0BTD0ZZ, 0BTD4ZZ, 0BTF0ZZ, 0BTF4ZZ, 0BTG0ZZ, 0BTG4ZZ, 0BTJ0ZZ, 0BTJ4ZZ, 0BTK0ZZ, 0BTK4ZZ, 0BTL0ZZ, 0BTL4ZZ, 0BTM0ZZ, 0BTM4ZZ, 0CTS0ZZ, 0CTS4ZZ, 0CTS7ZZ, 0CTS8ZZ	R06.8
	Devices	J95.00-J95.04, J95.09, Z43.0, Z93.0, Z99.0, J95.850, Z99.11, Z99.12, 0B110F4, 0B113F4, 0B114F4, 0B21XFZ, 0BHR0MZ, 0BHR3MZ,	N/A

Categories of CCCs			Corresponding LLCs Diagnosis ICD-10 (KCD-6)
**Categories**	**Subcategories**	**Diagnosis and Procedure codes**	
		0BHR4MZ, 0BHS0MZ, 0BHS3MZ, 0BHS4MZ, 0BW10FZ, 0BW13FZ, 0BW14FZ, 0JH604Z, 0JH634Z, 0JH804Z, 0JH834Z, 0WQ6XZ2, 3E1F78Z	
	Transplantation	T86.810, T86.811, T86.819, Z94.2, 0BYC0Z0, 0BYC0Z1, 0BYC0Z2, 0BYD0Z0, 0BYD0Z1, 0BYD0Z2, 0BYF0Z0, 0BYF0Z1, 0BYF0Z2, 0BYG0Z0, 0BYG0Z1, 0BYG0Z2, 0BYH0Z0, 0BYH0Z1, 0BYH0Z2, 0BYJ0Z0, 0BYJ0Z1, 0BYJ0Z2, 0BYK0Z0, 0BYK0Z1, 0BYK0Z2, 0BYL0Z0, 0BYL0Z1, 0BYL0Z2, 0BYM0Z0, 0BYM0Z1, 0BYM0Z2	N/A
**Renal & urologic**	Congenital anomalies	Q60-Q64	N04, N17, N18, N19, N25.8, Q60.1, Q60.6, Q61.4, Q61.9, Q64.2
	Chronic renal failure	N18	
	Other	Z90.5, Z90.6, 0T160Z8, 0T160ZA, 0T164Z8, 0T164ZA, 0T170Z8, 0T170ZA, 0T174Z8, 0T174ZA, 0T180Z8, 0T180ZA, 0T184Z8, 0T184ZA, 0TB60ZZ, 0TB63ZZ, 0TB64ZZ, 0TB67ZZ, 0TB68ZZ, 0TB70ZZ, 0TB73ZZ, 0TB74ZZ, 0TB77ZZ, 0TB78ZZ, 0TT00ZZ, 0TT04ZZ, 0TT10ZZ, 0TT14ZZ, 0TT20ZZ, 0TT24ZZ, 0TT60ZZ, 0TT64ZZ, 0TT67ZZ, 0TT68ZZ, 0TT70ZZ, 0TT74ZZ, 0TT77ZZ, 0TT78ZZ, 0TTB0ZZ, 0TTB4ZZ, 0TTB7ZZ, 0TTB8ZZ, 0TTD0ZZ, 0TTD4ZZ, 0TTD7ZZ, 0TTD8ZZ	
	Chronic bladder diseases	G83.4, N31.2, N31.9	
	Devices	T85.71XA, Z93.50-Z93.52, Z93.59, Z93.6, Z91.15, Z99.2, Z43.5, Z43.6, Z46.6, 031209D, 031209F, 03120AD, 03120AF, 03120JD, 03120JF, 03120KD, 03120KF, 03120ZD, 03120ZF, 031309D, 031309F, 03130AD, 03130AF, 03130JD, 03130JF, 03130KD, 03130KF, 03130ZD, 03130ZF, 031409D, 031409F, 03140AD, 03140AF, 03140JD, 03140JF, 03140KD, 03140KF, 03140ZD, 03140ZF, 031509D, 031509F, 03150AD, 03150AF, 03150JD, 03150JF, 03150KD, 03150KF, 03150ZD, 03150ZF, 031609D, 031609F, 03160AD, 03160AF, 03160JD, 03160JF, 03160KD, 03160KF, 03160ZD, 03160ZF, 031709D, 031709F, 03170AD, 03170AF, 03170JD, 03170JF, 03170KD, 03170KF, 03170ZD, 03170ZF, 031809D, 031809F, 03180AD, 03180AF, 03180JD, 03180JF, 03180KD, 03180KF, 03180ZD, 03180ZF, 031909F, 03190AF, 03190JF, 03190KF, 03190ZF, 031A09F, 031A0AF,	N/A

Categories of CCCs			Corresponding LLCs Diagnosis
Categories	Subcategories	Diagnosis and Procedure codes	ICD-10 (KCD-6)
		031A0JF, 031A0KF, 031A0ZF, 031B09F, 031B0AF, 031B0JF, 031B0KF, 031B0ZF, 031C09F, 031C0AF, 031C0JF, 031C0KF, 031C0ZF, 03WY0JZ, 03WY3JZ, 03WY4JZ, 03WYXJZ, 05HY33Z, 06HY33Z, 0JH60WZ, 0JH60XZ, 0JH63WZ, 0JH63XZ, 0JH80WZ, 0JH80XZ, 0JH83WZ, 0JH83XZ, 0JHD0WZ, 0JHD0XZ, 0JHD3WZ, 0JHD3XZ, 0JHF0WZ, 0JHF0XZ, 0JHF3WZ, 0JHF3XZ, 0JHL0WZ, 0JHL0XZ, 0JHL3WZ, 0JHL3XZ, 0JHM0WZ, 0JHM0XZ, 0JHM3WZ, 0JHM3XZ, 0T130ZB, 0T134ZB, 0T140ZB, 0T144ZB, 0T16079, 0T1607C, 0T1607D, 0T160J9, 0T160JC, 0T160JD, 0T160K9, 0T160KC, 0T160KD, 0T160Z9, 0T160ZC, 0T160ZD, 0T163JD, 0T16479, 0T1647C, 0T1647D, 0T164J9, 0T164JC, 0T164JD, 0T164K9, 0T164KC, 0T164KD, 0T164Z9, 0T164ZC, 0T164ZD, 0T17079, 0T1707C, 0T1707D, 0T170J9, 0T170JC, 0T170JD, 0T170K9, 0T170KC, 0T170KD, 0T170Z9, 0T170ZC, 0T170ZD, 0T173JD, 0T17479, 0T1747C, 0T1747D, 0T174J9, 0T174JC, 0T174JD, 0T174K9, 0T174KC, 0T174KD, 0T174Z9, 0T174ZC, 0T174ZD, 0T18079, 0T1807C, 0T1807D, 0T180J9, 0T180JC, 0T180JD, 0T180K9, 0T180KC, 0T180KD, 0T180Z9, 0T180ZC, 0T180ZD, 0T183JD, 0T18479, 0T1847C, 0T1847D, 0T184J9, 0T184JC, 0T184JD, 0T184K9, 0T184KC, 0T184KD, 0T184Z9, 0T184ZC, 0T184ZD, 0T1B0ZD, 0T1B4ZD, 0T25X0Z, 0T29X0Z, 0T29XYZ, 0T2BX0Z, 0T9000Z, 0T9030Z, 0T9040Z, 0T9070Z, 0T9080Z, 0T9100Z, 0T9130Z, 0T9140Z, 0T9170Z, 0T9180Z, 0T9370Z, 0T9380Z, 0T9470Z, 0T9480Z, 0TQ67ZZ, 0TQ77ZZ, 3E1K38Z, 3E1M39Z, 5A1D60Z	
	Transplantation	T86.10-T86.12, Z94.0, 0TY00Z0, 0TY00Z1, 0TY00Z2, 0TY10Z0, 0TY10Z1, 0TY10Z2	N/A
**Gastrointestinal**	Congenital anomalies	Q39.0-Q39.4, Q41-Q45	K55.0, K55.9, K72, K74, K76.5, K86.8, Q39.6, Q41.0, Q41.9, Q43.1, Q43.7, Q44.2, Q44.5, Q44.7
	Chronic liver disease and cirrhosis	K73, K74, K75.4, K760-K763, K765, K768	
	Inflammatory bowel diseases	K50, K51	
	Other	I82.0, K55.1, K56.2, K59.3, Z98.0, Z90.3, Z90.49, 0CT70ZZ, 0CT7XZZ,	

Categories of CCCs			Corresponding LLCs Diagnosis
**Categories**	**Subcategories**	**Diagnosis and Procedure codes**	**ICD-10 (KCD-6)**
		0D13079, 0D1307A, 0D1307B, 0D1607A, 0D160ZA, 0DT50ZZ, 0DT54ZZ, 0DT57ZZ, 0DT58ZZ, 0DT60ZZ, 0DT64ZZ, 0DT67ZZ, 0DT68ZZ, 0DT80ZZ, 0DT84ZZ, 0DT87ZZ, 0DT88ZZ, 0DT90ZZ, 0DT94ZZ, 0DT97ZZ, 0DT98ZZ, 0DTE0ZZ, 0DTE4ZZ, 0DTE7ZZ, 0DTE8ZZ, 0FT00ZZ, 0FT04ZZ, 0FTG0ZZ, 0FTG4ZZ	
	Devices	K94.20, K94.22, K94.23, K94.29, Z93.1-Z93.4, Z43.1-Z43.4, Z46.51, Z46.59, 0D11074, 0D110J4, 0D110K4, 0D110Z4, 0D113J4, 0D11474, 0D114J4, 0D114K4, 0D114Z4, 0D15074, 0D150J4, 0D150K4, 0D150Z4, 0D153J4, 0D15474, 0D154J4, 0D154K4, 0D154Z4, 0D16074, 0D160J4, 0D160J9, 0D160JA, 0D160K4, 0D160K9, 0D160KA, 0D160Z4, 0D163J4, 0D16474, 0D164J4, 0D164J9, 0D164JA, 0D164K4, 0D164K9, 0D164KA, 0D164Z4, 0D16874, 0D168J4, 0D168J9, 0D168JA, 0D168K4, 0D168K9, 0D168KA, 0D168Z4, 0D1B0Z4, 0D1B4Z4, 0D1B8Z4, 0D1H0Z4, 0D1H4Z4, 0D1H8Z4, 0D1K0Z4, 0D1K4Z4, 0D1K8Z4, 0D1L0Z4, 0D1L4Z4, 0D1L8Z4, 0D1N0Z4, 0D1N4Z4, 0D1N8Z4, 0D20X0Z, 0D20XUZ, 0D20XYZ, 0D787ZZ, 0D7E7ZZ, 0DBB7ZZ, 0DH50DZ, 0DH50UZ, 0DH53DZ, 0DH53UZ, 0DH54DZ, 0DH54UZ, 0DH57DZ, 0DH57UZ, 0DH58DZ, 0DH58UZ, 0DH63UZ, 0DH64UZ, 0DHA3UZ, 0DHA4UZ, 0DHA8UZ, 0DN87ZZ, 0DNE7ZZ, 0DW04UZ, 0DW08UZ, 0WQFXZ2, 3E1G78Z, 3E1H78Z	N/A
	Transplantation	T86.40-T86.42, T86.890, T86.891, T86.899, T86.850, T86.851, T86.859, Z94.4, Z94.82, Z94.83, 0DY80Z0, 0DY80Z1, 0DY80Z2, 0DYE0Z0, 0DYE0Z1, 0DYE0Z2, 0FY00Z0, 0FY00Z1, 0FY00Z2, 0FYG0Z0, 0FYG0Z1, 0FYG0Z2, 3E030U0, 3E030U1, 3E033U0, 3E033U1, 3E0J3U0, 3E0J3U1, 3E0J7U0, 3E0J7U1, 3E0J8U0, 3E0J8U1	N/A
**Hematologic or immunologic**	Hereditary anemias	D55-D58	B20-B24
	Aplastic anemias	D60-D61, D71	D56.1, D61.0, D61.9, D70, D76.1, D81, D82.1, D83, D89.1,
	Hereditary immunodeficiency	D80-D89, D72.0, M30.3, M35.9	E31.0,
	Coagulation/hemorrhagic	D66, D68.2, D69.41, D69.42, D69.49	

Categories of CCCs			Corresponding LLCs Diagnosis ICD-10 (KCD-6)
**Categories**	**Subcategories**	**Diagnosis and Procedure codes**	
	Leukopenia	D70.0, D70.4	M31.3, M32.1, M89.5
	Hemophagocytic Syndromes	D76.1-D76.3	
	Sarcoidosis	D86.9	
	Acquired immunodeficiency	B20-B24	
	Polyarteritis nodosa and related conditions	M30.0, M31.0, M31.1, M31.30, M31.4, M31.6	
	Diffuse diseases of connective tissue	M32.10, M33.90, M34.0, M34.1, M34.9	
	Other	07TP0ZZ, 07TP4ZZ	
	Devices	N/A	N/A
	Transplantation	07YP0Z0, 07YP0Z1, 07YP0Z2, 30230AZ, 30230G0, 30230G1, 30230X0, 30230X1, 30230Y0, 30230Y1, 30233AZ, 30233G0, 30233G1, 30233X0, 30233X1, 30233Y0, 30233Y1, 30240AZ, 30240G0, 30240G1, 30240X0, 30240X1, 30240Y0, 30240Y1, 30243AZ, 30243G0, 30243G1, 30243X0, 30243X1, 30243Y0, 30243Y1, 30250G0, 30250G1, 30250X0, 30250X1, 30250Y0, 30250Y1, 30253G0, 30253G1, 30253X0, 30253X1, 30253Y0, 30253Y1, 30260G0, 30260G1, 30260X0, 30260X1, 30260Y0, 30260Y1, 30263G0, 30263G1, 30263X0, 30263X1, 30263Y0, 30263Y1	N/A
**Metabolic**	Amino acid metabolism	E70.0, E70.2, E70.3, E70.4, E70.8, E71.0-E71.5, E72.0-E72.4, E72.8, E72.9	E34.8, E70.2, E71, E72, E74, E75, E76, E77, E79.1, E83.0, E88.0, E88.1,<br>H49.8
	Carbohydrate metabolism	E74.0-E74.4, E74.8, E74.9	
	Lipid metabolism	E75, E77.0, E77.1, E78.0-E78.4, E78.5-E78.9, E88.1, E88.8	
	Storage disorder	E76.0-E76.3, E85	
	Other metabolic disorders	277.4, E79.1, E79.8, E80.4-E80.7, E83.0, E83.1, E83.3, E83.4, D84.1, E88,	

Categories of CCCs			Corresponding LLCs Diagnosis ICD-10 (KCD-6)
Categories	Subcategories	Diagnosis and Procedure codes	
		H49.8	
	Endocrine disorders	E00.9, E23.0, E23.2, E22.2, E23.3, E23.7, E24.0, E24.2, E24.3, E24.8, E24.9, E26.81, E25.0, E25.8, E25.9, 0GT00ZZ, 0GT04ZZ, 0GT40ZZ, 0GT44ZZ, 0GTK0ZZ, 0GTK4ZZ, 0GTR0ZZ, 0GTR4ZZ, 0UT20ZZ, 0UT24ZZ, 0UT27ZZ, 0UT28ZZ, 0UT2FZZ, 0UT40ZZ, 0UT44ZZ, 0UT47ZZ, 0UT48ZZ, 0UT70ZZ, 0UT74ZZ, 0UT90ZZ, 0UT94ZZ, 0UT97ZZ, 0UT98ZZ, 0UT9FZZ, 0UTC0ZZ, 0UTC7ZZ, 0UTC8ZZ, 0VTC0ZZ, 0VTC4ZZ, 0W4M070, 0W4M0J0, 0W4M0K0, 0W4M0Z0, 0W4N071, 0W4N0J1, 0W4N0K1, 0W4N0Z1	
	Devices	Z46.81, Z96.41, 0JH60VZ, 0JH63VZ, 0JH70VZ, 0JH73VZ, 0JH80VZ, 0JH83VZ, 0JHD0VZ, 0JHD3VZ, 0JHF0VZ, 0JHF3VZ, 0JHG0VZ, 0JHG3VZ, 0JHH0VZ, 0JHH3VZ, 0JHL0VZ, 0JHL3VZ, 0JHM0VZ, 0JHM3VZ, 0JHN0VZ, 0JHN3VZ, 0JHP0VZ, 0JHP3VZ, 0JHT0VZ, 0JHT3VZ	N/A
	Transplantation	N/A	N/A
**Other congenital or genetic defect**	Chromosomal anomalies	Q90.9, Q91.3, Q91.4, Q91.7, Q92.8, Q93, Q95.0, Q96.9, Q97, Q98, Q99.8, Q99.9	P96.0,<br>Q74.3, Q74.8, Q75.0, Q77.2, Q77.3,<br>Q77.4, Q78.0, Q78.5, Q79.2, Q79.3,<br>Q80.4, Q81, Q82.1, Q82.4, Q86.0, Q87.0, Q87.1, Q87.2, Q87.8, Q91, Q92.0, Q92.1, Q92.4, Q92.7, Q92.8,<br>Q93.2, Q93.3, Q93.4, Q93.5, Q93.8,<br>Q95.2, Q99.2,
	Bone and joint anomalies	E34.3, M41.0, M41.2, M41.30, M41.8, M41.9, M43.30, M96.5, Q72.2, Q75.0, Q75.2, Q75.9, Q76.0-Q76.2, Q76.4-Q76.7, Q77, Q78.0-Q78.4, Q78.8, Q78.9	
	Diaphragm and abdominal wall	K44.9, Q79.0-Q79.5, Q79.9, Q79.59	
	Other congenital anomalies	Q81, Q87.1-Q87.3, Q87.40, Q87.81, Q87.89, Q89.7, Q89.9, Q99.2	
**Malignancy**	Neoplasms	C00-C96, D01-D09, D3A.0, D37-D49, Q85.0, 3E00X05, 3E01305, 3E02305, 3E03005, 3E03305, 3E04005, 3E04305, 3E05005, 3E05305, 3E06005, 3E06305, 3E0A305, 3E0F305, 3E0F705, 3E0F805, 3E0G305, 3E0G705,	C00-C97

Categories of CCCs			Corresponding LLCs Diagnosis ICD-10 (KCD-6)
Categories	Subcategories	Diagnosis and Procedure codes	
		3E0G805, 3E0H305, 3E0H705, 3E0H805, 3E0J305, 3E0J705, 3E0J805, 3E0K305, 3E0K705, 3E0K805, 3E0L305, 3E0L705, 3E0M305, 3E0M705, 3E0N305, 3E0N705, 3E0N805, 3E0P305, 3E0P705, 3E0P805, 3E0Q305, 3E0Q705, 3E0R305, 3E0S305, 3E0V305, 3E0W305, 3E0Y305, 3E0Y705	D33, D43, D44.4, D48<br>T86.0
	Devices	N/A	N/A
	Transplantation	T86.00-T86.02, T86.09, Z94.81, Z94.84	N/A
**Premature & neonatal**	Fetal malnutrition	P05.01, P05.11, P05.02, P05.12, P05.2, P05.9	P10.1, P11.2, P20.0, P20.1, P21.0, P21.9, P28.5, P35.0, P35.1, P35.8, P37.1, P52.4, P52.5, P52.9, P83.2, P91.2, P91.6
	Extreme immaturity	P07.01, P07.02, P07.21-P07.25	
	Cerebral hemorrhage at birth	P10.0, P10.1, P10.4, P52.4, P52.8	
	Spinal cord injury at birth	P11.5	
	Birth asphyxia	P21.0, P21.9, P84	
	Respiratory diseases	P25.0-P25.3, P25.8, P27.0, P27.1, P27.8	
	Hypoxic-ischemic encephalopathy	P91.6	
	Other	P35.0, P35.1, P25.21, P25.22, P56.0, P57.0, P57.8, P61.3, P61.4, P77.3, P83.2, P91.2	
**Miscellaneous**		N/A	H11.1<br>Q85.8<br>Z51.5
	Devices	T84.019A, T84.029A, T84.039A, T84.049A, T84.059A, T84.069A, T84.099A, T84.498A, T84.119A, T84.129A, T84.199A, T84.498A, T84.50XA, T84.60XA, , T84.7XXA, T86.90-T86.92, T86.99, T86.10-T86.12, T86.40-T86.42, T86.20-T86.22, T86.810, T86.811, T86.819, T86.00-T86.02, T86.09, T86.890, T86.891, T86.899, T86.850, T86.851, T86.859, T86.5, T86.890, T86.891, T86.899, T87.0X9, T87.1X9, T87.2, Y83.1, Y83.3, Z99.81, 0RG00J0,	

Categories of CCCs			Corresponding LLCs Diagnosis ICD-10 (KCD-6)
**Categories**	**Subcategories**	**Diagnosis and Procedure codes**	
		0RG00J1, 0RG00JJ, 0RG00K0, 0RG00K1, 0RG00KJ, 0RG00Z0, 0RG00Z1, 0RG00ZJ, 0RG03J0, 0RG03J1, 0RG03JJ, 0RG03K0, 0RG03K1, 0RG03KJ, 0RG03Z0, 0RG03Z1, 0RG03ZJ, 0RG04J0, 0RG04J1, 0RG04JJ, 0RG04K0, 0RG04K1, 0RG04KJ, 0RG04Z0, 0RG04Z1, 0RG04ZJ, 0RG10J0, 0RG10J1, 0RG10JJ, 0RG10K0, 0RG10K1, 0RG10KJ, 0RG10Z0, 0RG10Z1, 0RG10ZJ, 0RG13J0, 0RG13J1, 0RG13JJ, 0RG13K0, 0RG13K1, 0RG13KJ, 0RG13Z0, 0RG13Z1, 0RG13ZJ, 0RG14J0, 0RG14J1, 0RG14JJ, 0RG14K0, 0RG14K1, 0RG14KJ, 0RG14Z0, 0RG14Z1, 0RG14ZJ, 0RG40J0, 0RG40J1, 0RG40JJ, 0RG40K0, 0RG40K1, 0RG40KJ, 0RG40Z0, 0RG40Z1, 0RG40ZJ, 0RG43J0, 0RG43J1, 0RG43JJ, 0RG43K0, 0RG43K1, 0RG43KJ, 0RG43Z0, 0RG43Z1, 0RG43ZJ, 0RG44J0, 0RG44J1, 0RG44JJ, 0RG44K0, 0RG44K1, 0RG44KJ, 0RG44Z0, 0RG44Z1, 0RG44ZJ, 0RG60J0, 0RG60J1, 0RG60JJ, 0RG60K0, 0RG60K1, 0RG60KJ, 0RG60Z0, 0RG60Z1, 0RG60ZJ, 0RG63J0, 0RG63J1, 0RG63JJ, 0RG63K0, 0RG63K1, 0RG63KJ, 0RG63Z0, 0RG63Z1, 0RG63ZJ, 0RG64J0, 0RG64J1, 0RG64JJ, 0RG64K0, 0RG64K1, 0RG64KJ, 0RG64Z0, 0RG64Z1, 0RG64ZJ, 0RGA0J0, 0RGA0J1, 0RGA0JJ, 0RGA0K0, 0RGA0K1, 0RGA0KJ, 0RGA0Z0, 0RGA0Z1, 0RGA0ZJ, 0RGA3J0, 0RGA3J1, 0RGA3JJ, 0RGA3K0, 0RGA3K1, 0RGA3KJ, 0RGA3Z0, 0RGA3Z1, 0RGA3ZJ, 0RGA4J0, 0RGA4J1, 0RGA4JJ, 0RGA4K0, 0RGA4K1, 0RGA4KJ, 0RGA4Z0, 0RGA4Z1, 0RGA4ZJ, 0SG00J0, 0SG00J1, 0SG00JJ, 0SG00K0, 0SG00K1, 0SG00KJ, 0SG00Z0, 0SG00Z1, 0SG00ZJ, 0SG03J0, 0SG03J1, 0SG03JJ, 0SG03K0, 0SG03K1, 0SG03KJ, 0SG03Z0, 0SG03Z1, 0SG03ZJ, 0SG04J0, 0SG04J1, 0SG04JJ, 0SG04K0, 0SG04K1, 0SG04KJ, 0SG04Z0, 0SG04Z1, 0SG04ZJ	
	Transplantation	T86.5, T86.90-T86.92, T86.99, T86.890, T86.891, T86.899	N/A

CCCs: Complex chronic conditions; LLCs: Life-limiting conditions; ICD-10; International Classification of Disease, 10th revision; KCD: Korean Standard Classification of Diseases; CNS: Central nervous system; N/A: not applicable.

Supplementary Table S2. Demographic characteristics and pattern of healthcare utilization of children and young people with life-limiting conditions, 2015

Unit: number (%), mean ± SD

Variables	Total	Inpatient resource use		OPD use[b]	ED use[c]
		≥1 Hospitalization[a]	Length of stay		
Total	133,177	36,054 (27.1)	27.2±51.5	10.8±25.9	1.2±1.9
Age group (year)					
<1	9,084	6,042 (66.5)	25.7±34.4	4.8±8.1	1.4±1.4
1-9	35,247	11,236 (31.9)	33.1±56.7	16.2±32.9	1.5±2.3
10-19	49,120	10,509 (21.4)	25.4±53.1	11.2±26.7	1.1±0.8
20-24	39,726	8,267 (20.8)	22.5±51.6	6.5±16.9	1.2±2.4
Level of income					
High	46,137	11,088 (24.0)	25.2±48.0	10.4±25.1	1.2±1.4
Medium	53,547	16,183 (30.2)	26.4±48.4	10.0±23.9	1.2±1.2
Low	33,493	8,783 (26.2)	31.2±60.4	12.8±29.6	1.3±2.9
Area of residence					
Metropolitan	60,322	16,231 (26.9)	27.1±51.1	11.8±28.4	1.2±2.4
Non-metropolitan	72,855	19,823 (27.2)	27.3±51.8	10.1±23.7	1.2±1.1
Diagnostic group					
Cancer	34,943	10,596 (30.3)	30.7±51.0	9.5±19.0	1.6±1.9
Non-cancer	98,234	25,458 (25.9)	25.7±51.7	11.4±28.1	1.2±1.9
Length of stay (days)					
<30		28,435 (79.2)	8.6±6.8	18.6±31.6	1.6±2.4
30-89		4,671 (13.0)	52.6±16.9	32.8±38.2	1.9±2.1
90-119		866 (2.4)	104.1±8.6	38.3±37.3	2.4±3.1
>120		1,940 (5.4)	206.8±79.4	29.4±31.0	2.1±2.2

SD: standard deviation; OPD: outpatient department; ED: emergency department.

[a] No. of patients who were ever hospitalized

[b] No. of patients who ever visited OPD

[c] No. of patients who ever visited ED

Supplementary Table S3. Patterns of healthcare utilization and expenditure in the year prior to death among deceased children and young people aged 0 to 24 years with life-limiting conditions, by age group, 2015

Unit: number (%), mean±SD

Variables	Total	Age of death (year)				*p*-value
		<1	1-9	10-19	20-24	
Total	3,995	1,137 (28.5)	781 (19.5)	1,172 (29.3)	905 (22.7)	
Total mean expenditure	43,819±51,417	49,839±52,879	44,998±48,769	42,827±53,724	36,522±47,689	<0.001
Inpatient service						
≥1 Hospitalization per year[a]	3,654 (91.5)	1,124 (98.9)	716 (91.7)	1,028 (87.7)	786 (86.9)	
Length of stay (days)	99.2±100.0	79.3±81	112.5±105.8	103.9±103.6	109.3±109.5	<0.001
Income expenditure	41,563±50,347	49,389±52,898	42,294±48,279	39,645±52,009	33,582±44,967	<0.001
Outpatient service						
≥1 OPD visit per year[b]	3,318 (83.1)	565 (49.7)	761 (97.4)	1,137 (97.0)	855 (94.5)	
Per capita OPD visits	39.1±38.5	25.0±31.5	58.8±46.6	36.5±31.5	34.5±36.7	<0.001
Outpatient expenditure	2,125±6,279	384±1,420	2,552±3,803	3,027±8,781	2,778±7,338	<0.001
Emergency service						
≥1 ED visit per year[c]	1,517 (38.0)	245 (21.5)	373 (47.8)	505 (43.1)	394 (43.5)	
Per capita ED visits	2.0±3.4	1.6±1.0	2.2±3.4	1.9±2.5	2.3±5.0	0.085
Emergency expenditure	131±312	67±170	152±331	155±380	162±323	<0.001

SD: standard deviation; OPD: outpatient department; ED: emergency department.

All expenditure values are in 2019 US dollar: 1 USD = 1,121.10 KR.

[a] No. of patients who were ever hospitalized.

[b] No. of patients who ever visited OPD.

[c] No. of patients who ever visited ED.

Supplementary Table S4. Patterns of healthcare utilization and expenditure in the year prior to death among deceased children and young people aged 0 to 24 years with life-limiting conditions, by level of income

Unit: number (%), mean±SD

Variables	Total	Level of Income			*p*-value
		High	Medium	Low	
Total	3,995	1,114 (27.9)	1,732 (43.4)	1,149 (28.8)	
Total mean expenditure	43,819±51,417	46,121±52,704	44,688±52,450	40,276±48,351	0.017
Inpatient service					
≥1 Hospitalization per year[a]	3,654 (91.5)	1,009 (90.6)	1,608 (92.8)	1,037 (90.3)	
Length of stay (days)	99.2±99.9	99.3±97.4	89.3±91.1	114.4±12.9	<0.001
Inpatient expenditure	41,563±50,347	43,705±51,979	42,908±52,158	37,458±45,536	0.004
Outpatient service					
≥1 OPD visit per year[b]	3,318 (83.1)	945 (84.8)	1,350 (77.9)	1,023 (89.0)	
Per capita OPD visits	39.1±38.5	40.6±38.1	38.1±36.6	39.2±41.2	0.316
Outpatient expenditure	2,125±6,279	2,284±3,976	1,654±4,056	2,682±9,816	<0.001
Emergency service					
≥1 ED visit per year[c]	1,517 (38.0)	410 (36.8)	644 (37.2)	463 (40.3)	
Per capita ED visits	2.0±3.4	2.1±3.5	1.9±2.4	2.1±4.4	0.489
Emergency expenditure	131±312	133±325	126±300	136±315	0.682

SD: standard deviation; OPD: outpatient department; ED: emergency department.

All expenditure values are in 2019 US dollar: 1 USD = 1,121.10 KRW.

[a] No. of patients who were ever hospitalized.

[b] No. of patients who ever visited OPD.

[c] No. of patients who ever visited ED.

Supplementary Table S5. Residence and location of final hospital admission in the year prior to death among deceased children and young people aged 0 to 24 years with life-limiting conditions, 2013-2015

Unit: number or (%)

Residence of the deceased	Total	Location of final hospital admission before death: Seoul	Busan	Daegu	Incheon	Gwangju	Daejeon	Ulsan	Sejong	Gyeonggi	Gangwon	Chungbuk	Chung-nam	Jeonbuk	Jeonnam	Gyeong-buk	Gyeong-nam	Jeju	RI (%)
Total	3,995	1567	275	268	139	104	164	85	0	536	100	75	94	127	109	72	223	57	N/A
Seoul	674	604	3	4	3	0	2	0	0	44	5	1	1	1	2	1	2	1	89.6
Busan	273	30	190	4	0	0	1	2	0	2	0	1	0	1	0	2	40	0	69.6
Daegu	221	41	4	164	1	0	1	1	0	4	0	1	0	0	0	3	1	0	74.2
Incheon	214	61	0	0	114	1	0	0	0	33	3	0	1	1	0	0	0	0	53.3
Gwangju	107	20	0	0	0	64	0	1	0	1	0	0	0	1	19	0	0	1	59.8
Daejeon	162	43	0	1	0	1	112	0	0	2	0	1	2	0	0	0	0	0	69.1
Ulsan	117	15	10	3	1	0	1	73	0	0	0	0	0	0	0	1	13	0	62.4
Sejong	11	3	1	0	0	0	4	0	0	1	0	2	0	0	0	0	0	0	N/A
Gyeonggi	919	448	5	3	12	1	4	0	0	403	3	2	19	6	3	4	5	1	43.9
Gangwon	131	37	0	0	1	0	0	0	0	9	81	1	0	0	0	1	0	1	61.8
Chungbuk	127	46	1	3	1	0	8	0	0	3	0	63	2	0	0	0	0	0	49.6
Chungnam	190	70	1	4	3	0	24	0	0	11	1	1	67	5	1	1	1	0	35.3
Jeonbuk	159	37	1	0	1	4	2	0	0	3	1	0	1	109	0	0	0	0	68.6
Jeonnam	163	33	0	0	0	31	1	1	0	5	0	1	0	3	84	1	1	2	51.5
Gyeongbuk	198	29	9	75	2	1	3	3	0	9	4	1	1	0	0	57	4	0	28.8
Gyeongnam	260	33	49	7	0	1	1	4	0	5	2	0	0	0	0	1	156	1	60.0
Jeju	69	17	1	0	0	0	0	0	0	1	0	0	0	0	0	0	0	50	72.5

RI: Relevance index; N/A: not applicable.
